# Prospective analysis of clinically significant prostate cancer detection with [^18^F]DCFPyL PET/MRI compared to multiparametric MRI: a comparison with the histopathology in the radical prostatectomy specimen, the ProStaPET study

**DOI:** 10.1007/s00259-021-05604-9

**Published:** 2021-11-02

**Authors:** Yves J. L. Bodar, Ben G. J. C. Zwezerijnen, Patrick J. van der Voorn, Bernard H. E. Jansen, Ruth S. Smit, Sabrine Q. Kol, Dennie Meijer, Katelijne de Bie, Maqsood Yaqub, Bert A. D. Windhorst, Harry N. H. Hendrikse, André N. Vis, Daniela E. Oprea-Lager

**Affiliations:** 1 Department of Urology, Amsterdam University Medical Centres (VU University Medical Center), Amsterdam, the Netherlands; 2 Department of Radiology & Nuclear Medicine, Amsterdam University Medical Centres (VU University Medical Center), Amsterdam, the Netherlands; 3 Prostate Cancer Network, Amsterdam, the Netherlands; 4 Department of Pathology, Amsterdam University Medical Centres (VU University Medical Center), Amsterdam, the Netherlands; 5 Department of Clinical Pharmacology and Pharmacy, Amsterdam University Medical Centres (VU University Medical Center), Amsterdam, the Netherlands

**Keywords:** ^18^F-DCFPyL PET/MRI, Primary detection, Prostate cancer, PSMA, Targeted biopsy

## Abstract

**Purpose:**

Multiparametric magnetic resonance imaging (mpMRI) is a well-established imaging method for localizing primary prostate cancer (PCa) and for guiding targeted prostate biopsies. [^18^F]DCFPyL positron emission tomography combined with MRI (PSMA-PET/MRI) might be of additional value to localize primary PCa. The aim of this study was to assess the diagnostic performance of [^18^F]DCFPyL-PET/MRI vs. mpMRI in tumour localization based on histopathology after robot-assisted radical-prostatectomy (RARP), also assessing biopsy advice for potential image-guided prostate biopsies.

**Methods:**

Thirty prospectively included patients with intermediate to high-risk PCa underwent [^18^F]DCFPyL-PET/MRI and mpMRI prior to RARP. Two nuclear medicine physicians and two radiologists assessed tumour localization on [^18^F]DCFPyL-PET/MRI and on mpMRI respectively, and gave a prostate biopsy advice (2 segments) using a 14-segment model of the prostate. The uro-pathologist evaluated the RARP specimen for clinically significant PCa (csPCa) using the same model. csPCa was defined as any PCa with Grade Group (GG) ≥ 2. The biopsy advice based on imaging was correlated with the final histology in the RARP specimen for a total-agreement analysis. An additional near-agreement correlation was performed to approximate clinical reality.

**Results:**

Overall, 142 of 420 (33.8%) segments contained csPCa after pathologic examination. The segments recommended for targeted biopsy contained the highest GG PCa segment in 27/30 patients (90.0%) both for [^18^F]DCFPyL-PET/MRI and mpMRI. Areas under the receiver operating characteristics curves (AUC), sensitivity, specificity, positive predictive value (PPV), and negative predictive value (NPV) for the total-agreement detection of csPCa per segment using [^18^F]DCFPyL-PET/MRI were 0.70, 50.0%, 89.9%, 71.7%, and 77.9%, respectively. These results were 0.75, 54.2%, 94.2%, 82.8%, and 80.1%, respectively, for mpMRI only.

**Conclusion:**

Both [^18^F]DCFPyL-PET/MRI and mpMRI were only partly able to detect csPCa on a per-segment basis. An accurate detection (90.0%) of the highest GG lesion at patient-level was observed when comparing both [^18^F]DCFPyL-PET/MRI and mpMRI biopsy advice with the histopathology in the RARP specimen. So, despite the finding that [^18^F]DCFPyL-PET/MRI adequately detects csPCa, it does not outperform mpMRI.

**Supplementary Information:**

The online version contains supplementary material available at 10.1007/s00259-021-05604-9.

## Introduction

The diagnostic work-up of patients with an elevated prostate-specific antigen (PSA)-level and an increased risk of prostate cancer (PCa) is continuously changing [1]. Histopathological verification is required to confirm PCa diagnosis and is standardly attained through systematic ultrasound-guided prostate biopsies [2]. In recent years, the addition of multiparametric magnetic resonance imaging (mpMRI) of the prostate before prostate biopsy has been implemented in the guidelines by the European Association of Urology (EAU) [1]. In this systematic approach, prostate biopsies need to be combined with mpMRI-targeted prostate biopsies (MRI-TBx) of radiologically abnormal regions within the prostate [3–5]. As a result, mpMRI assists in improving the yield of prostate biopsies for the diagnosis of clinically significant PCa (csPCa), and besides enhances local staging of PCa [6]. In this, mpMRI provides information on the suspicion of extracapsular extension (ECE) and invasion into the seminal vesicles (SVI) [7]. Thus, mpMRI is essential in determining the feasibility of radical surgery, in guiding surgical planning, such as whether nerve sparing surgery is appropriate, and in determining whether concomitant pelvic lymph node dissection is indicated [6, 8, 9]. Similarly, for planning EBRT, local staging is crucial for deciding on radiation dose and adjuvant therapies [6, 10].

Recently, prostate-specific membrane antigen (PSMA) binding positron emission tomography (PET) tracers have been developed. PSMA expression is associated with higher PCa tumour grades and an increased risk of disease progression [11]. So far, most studies have used ^68^ Ga-labelled PSMA tracers to detect biochemically recurrent disease—with excellent results [12, 13]. Apart from ^68^ Ga-labelled PSMA tracers, ^18^F-labelled tracers such as [^18^F]DCFPyL are available. These tracers have, in theory, technological advantages over ^68^ Ga-labelled PSMA tracers, providing a higher spatial resolution next to a longer half-life, which may result in a more accurate staging due to the detection of small local tumour deposits[12].

PET acquisitions are typically combined with computed tomography (CT) for attenuation correction and anatomical correlation. However, several studies with ^68^ Ga-labelled PSMA tracers found that combining PSMA-PET with MRI improved detection of local PCa and metastases [14, 15]. Additionally, PSMA-PET/MRI enables whole-body staging at a lower radiation dose, and can guide prostate biopsies in a single hospital visit, which is advantageous over performing both a pre-biopsy mpMRI and a staging PSMA PET/CT.

This is the largest prospective study investigating the value of PSMA-PET using [^18^F]DCFPyL combined with MRI compared to that of mpMRI alone [16, 17]. In this, we compared tumour localization and local tumour staging of both imaging modalities to the gold standard, i.e., tumour localization and tumour stage determined in the radical prostatectomy specimen. Finally, we assessed whether the advice of the potential location of the targeted biopsies was different between [^18^F]DCFPyL PET combined with MRI to that of mpMRI alone.

## Methods

### Study design and patient population

**Fig. 1:**
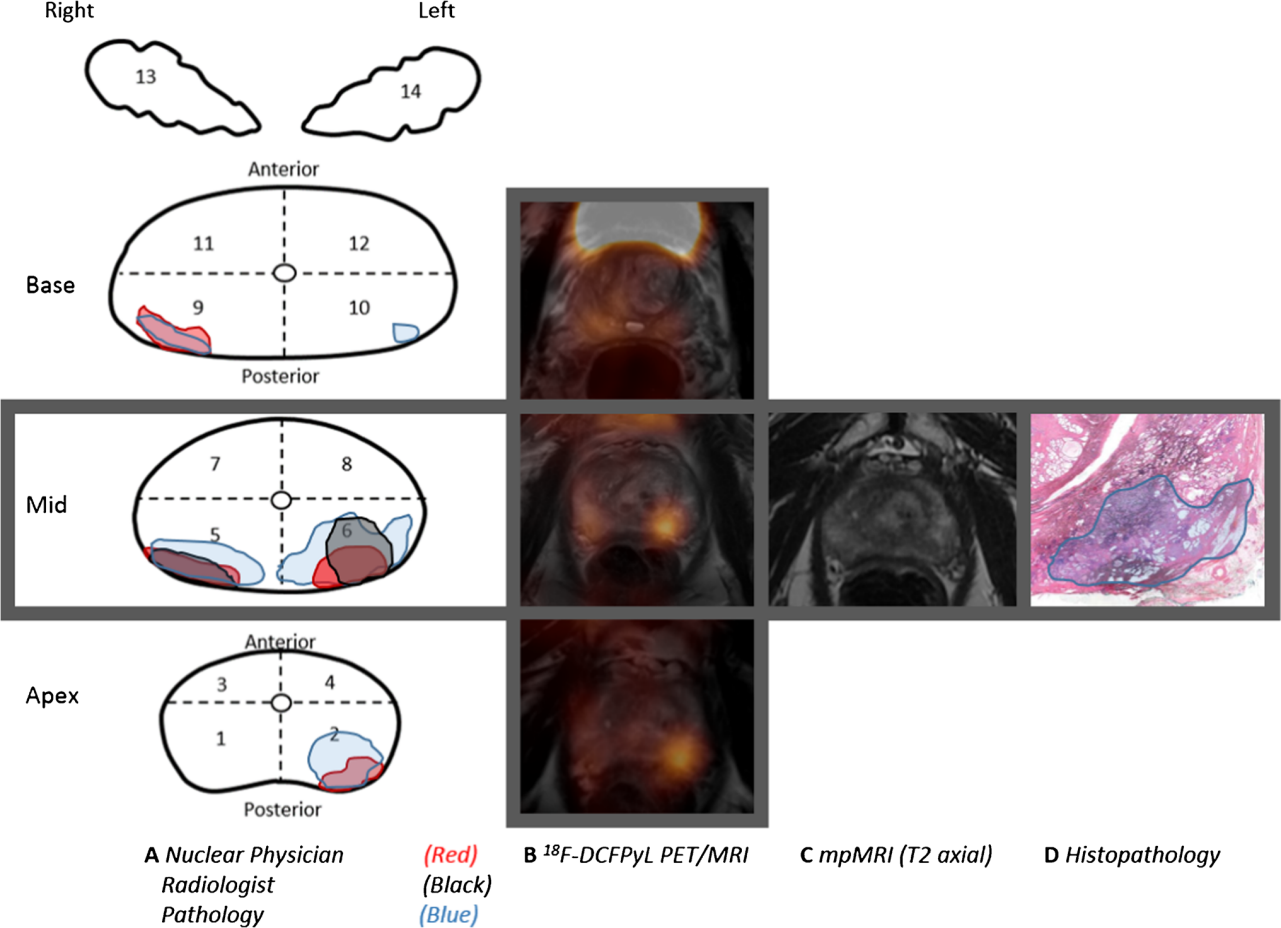
A 66-year-old patient with a biopsy-proven PCa with a GG of 3 and an iPSA of 19.0 ng/mL. (A) Schematic drawing of the 14 prostate segment mapping model included in the study and used in the radical prostatectomy specimen; (B) fused [^18^F]PSMA PET/MRI images show intense focal uptake in the left posterior side of the mid-prostate. Based on the highest SUV_max_ of 3.93 and tumour size, segment 6 was advised for targeted biopsy. (C) mpMRI T2 axial images show an PI-RADS 4 index lesion on the left posterior side of the mid-prostate, which was also advised for targeted biopsy. (D) Histopathology shows a similar tumour focus in segment 6, with a GG 3 PCa with extraprostatic involvement (pT3a), hereby confirming the index lesion localization

This was a prospective, observational, non-randomized cohort study. Patients were included from January 2018 until January 2021 at the Amsterdam University Medical Center. Ethical approval was gained from the local institutional review board (review VUmc number 2017.498), and all patients signed written informed consent to be included in this study. Patients who had histologically proven PCa, and who were planned to receive a mpMRI and [^18^F]DCFPyL PET/MRI for primary staging were included. All patients underwent RARP after mpMRI and [^18^F]DCFPyL PET/MRI. Collected clinical parameters included age, prostate volume as measured on trans-rectal ultrasound, initial prostate-specific antigen (PSA)-level, clinical T-stage, pathological biopsy features (histopathological grade, number of cores with cancer), and European Association of Urology (EAU)-risk category [2, 18]. To localize and characterize the prostatic tumours in a standardized manner, a 12-segment anatomic mapping model of the prostate was used, with 2 additional segments representing the seminal vesicles (Fig. 1) [19].

### Imaging protocol

[^18^F]DCFPyL PET/MRI images were obtained using a 3.0-T Ingenuity TF PET/MRI system (Philips Medical Systems, Cleveland, Ohio, USA). [^18^F]DCFPyL was synthesized under Good Manufacturing Practices conditions with > 95% radiochemical purity and > 5 GBq/µmol molar activity [20]. Patients were administered a median dose of 310.2 MBq (interquartile range (IQR) 300.6–317.3) [^18^F]DCFPyL. Patients were scanned at a median of 123 min (IQR 117–132) post-injection. The protocol included a survey MRI for defining the scan trajectory acquisition (mid-femur to the basal field of the lungs). Attenuation correction of the subsequent PET scan was achieved by a vendor-dedicated MR sequence (atMR), which segments the image into two tissue classes (i.e., air, soft tissue). The following sequences were acquired: total body: coronal T1-weighted fast spin-echo (FSE), coronal short-tau inversion recovery (STIR), axial Dixon sequences; prostatic region: T2-weighted turbo spin-echo (TSE) and axial diffusion-weighted sequences. Next, a whole-body PET scan was performed, on average consisting of 6 bed positions, each with a 5-min acquisition time. Reconstructions included both 2-mm slices for visual interpretation and 4 mm for semi-quantitative purposes (matrix size 144 × 144, slice thickness 4 mm; matrix size 288 × 288, slice thickness 2 mm).

On the same day as the [^18^F]DCFPyL PET/MRI, a separate mpMRI was performed, following the recommendations of the Prostate Imaging and Reporting and Data System (PI-RADS) v2[21]. A 3-T field strength was used. Imaging protocol entails T2-weighted, high spatial resolution, anatomical imaging in sagittal, coronal, axial, and TSE orientation; diffusion-weighted imaging (DWI). Both scans were performed at a median of 27 days (IQR 14–34) before surgery.

### Image interpretation and biopsy advice

[^18^F]DCFPyL PET/MRI images were assessed by two independent nuclear medicine physicians with ample experience in reporting prostate PSMA-PET/CT and PET/MRI (DO, GZ), and the final report was drawn up in consensus. [^18^F]DCFPyL PET/MRI images were reviewed according to clinical routine and according to the E-PSMA guidelines (tumour detection in prostate; local advancement; lymph node/ bone metastases), blinded by histopathological and mpMRI results [22, 23]. Additionally, PCa localization was reported using the 14-segment mapping model [19] (Fig. 1). Similar to the PI-RADS v2 classification for mpMRI, for every prostate segment, a 5-point PSMA-RADS Likert scale was deployed for PET (1 = very low (clinically significant cancer is highly unlikely to be present); 2 = low (clinically significant cancer is unlikely to be present); 3 = intermediate (the presence of clinically significant cancer is equivocal); 4 = high (clinically significant cancer is likely to be present); 5 = very high (clinically significant cancer is highly likely to be present). Lastly, the index lesion was identified and mapped on the 14-segment model. For multifocal PCa without extracapsular extension, the index lesion was defined as the lesion suspected of having both the highest GG and the largest tumour volume. For multifocal PCa with suspicion of extracapsular extension, the index lesion was the lesion suspected of extracapsular extension [24]. For (semi)quantitative analysis, a *volume of interest* (VOI) was manually drawn onto suspicious lesions using Intellispace Portal (Philips®, the Netherlands/USA). For each lesion detected on [^18^F]DCFPyL PET/MRI, the SUV_max_ was retrieved, and defined as the maximum SUV value within the VOI [25].

For mpMRI, images were independently reviewed according to clinical routine (PI-RADS v2; local advancement) by two radiologists with specific experience in prostate imaging (RS, SK). The mpMRI readers were blinded for the histopathological and [^18^F]DCFPyL PET/MRI results, and the final report was drawn up in consensus. PCa localization was reported using the 14-segment model, according to the PI-RADS v2 5-point rating scale [19]. The radiologists identified the index lesion according to the same criteria as the nuclear medicine physicians.

### Pathology analysis

After RARP, the prostate specimens were formalin-fixed and paraffin-embedded, photographed, and inked in 4 quadrants. Sections of 4 mm were cut perpendicular on the urethra and all sections were sliced with the proper tool into 3-μm slices upon staining with haematoxylin and eosin (H&E). An experienced uropathologist (PV) reviewed the slices according to clinical protocol and was blinded to the imaging results. In these specimens, the following histopathologic features were assessed: GG according to International Society of Urological Pathology (ISUP) protocols and pathological T-stage according to the American Joint Committee on Cancer (AJCC) TNM 8^th^ edition [6, 26]. Additionally, tumour locations were mapped using the aforementioned 14-segment model [19]. For all separate segments with PCa, GG was provided with a notification of the presence of ECE (pT3a). Clinically significant PCa (csPCa) was defined as PCa with a GG ≥ 2 or any tumour with ≥ pT3a. For each patient, the segment with the index lesion was noted separately. The index lesion was defined as the lesion with the highest GG or stage within the prostate.

### Statistical analysis

The suspected PCa locations detected with [^18^F]DCFPyL PET/MRI and mpMRI, respectively, were compared to final histology of the RARP specimen. The sensitivity, specificity, positive predictive value (PPV), and the negative predictive value (NPV) for detecting csPCa on a per-segment basis were calculated for both [^18^F]DCFPyL PET/MRI and mpMRI based on the 14-segment model of the prostate. As no anatomical landmarks are present in the prostate to delineate the 14 segments, artificial segmentation can occur. This artificial segmentation can cause a discrepancy between imaging and histopathological findings, while both are referring to the same lesion. To overcome this, a second analysis of diagnostic accuracy was conducted, in which [^18^F]DCFPyL PET/MRI and mpMRI findings were considered correlated with csPCa even when there was a discrepancy of up to 1 segment in the coronal or sagittal plane, as previously described [27, 28]. This second analysis was labelled as *near-agreement*, while the *total-agreement* analysis represents the direct segmental comparison without leniency. In addition, the sensitivity, specificity, PPV, and NPV of [^18^F]DCFPyL PET/MRI and mpMRI were calculated for detecting extracapsular disease (≥ T3) on a patient level.

Differences in diagnostic performance (sensitivity, specificity) between [^18^F]DCFPyL PET/MRI and mpMRI were assessed for statistical significance using the McNemar test. Inter-reader variability for both [^18^F]DCFPyL PET/MRI and mpMRI was reported using proportional agreement scores for the detection of PCa and local staging. Agreement scores were defined as the proportion of total positive and/or negative findings that were similar for both readers as a proportion of the total findings. Agreement scores were presented as the proportion of overall agreement scores, positive agreement scores, and negative agreement scores [29]. Numerical variables were summarized with median values and interquartile ranges (IQR); categorical variables with proportions (%). To compare medians of non-parametric data, the Mann–Whitney-Wilcoxon test and the Kruskal–Wallis test were used (significance set at *p* < 0.05). Statistical analysis was performed with IBM® SPSS® Statistics for Windows®, version 26.

## Results

### Patient characteristics

A total of 30 patients was included in this study, and all patients were scheduled for RARP after [^18^F]DCFPyL PET/MRI and same-day mpMRI. Included patients had a median age of 69 years (IQR 66–73), and a median initial PSA level of 7.6 ng/mL (IQR 6.5–10.1). Following EAU guidelines, 16/30 (53.3%) patients had intermediate-risk PCa and 14/30 (46.6%) had high-risk PCa [3]. Pre- and post-operative characteristics of included patients are listed in Table 1. A total of 22/30 (73.3%) patients received an ePLND alongside the RALP. After surgical excision of a total of 307 lymph nodes, 2/22 (9.1%) patients had positive lymph node status. For all patients, mpMRI did not detect any pathologically enlarged lymph nodes within the scanning window. On [^18^F]DCFPyL PET/MRI, 2 patients had a suspicion of local N1 lymph node metastases, of which 1 had positive lymph nodes after ePLND. One patient that had both a negative mpMRI and [^18^F]DCFPyL PET/MRI for lymph node metastases did have a positive lymph node metastasis after ePLND.

**Table 1:** Pre- and postoperative characteristics of 30 patients undergoing [^18^F]DCFPyL-PET/MRI and mpMRI before robot-assisted radical prostatectomy

Baseline (pre-operative) characteristics			
		*Median*	*IQR*
*Age (years)*		69	66–73
*Prostate volume (mL)*		40	35–48
*Initial PSA (ng/mL)*		7.6	6.5–10.1
*Positive biopsy cores (% of total cores)*		30.4	10.0–12.8
		*n*	%
*Grade group (ISUP)*^a^	*1*	2	6.6
	*2*	4	13.3
	*3*	12	40.0
	*4*	6	20.0
	*5*	6	20.0
	*Total*	30	100.0
*Clinical* *T-stage*	*1c*	15	50.0
	*2a/b*	12	40.0
	*2c*	3	10.0
	*3a*	0	0.0
	*Total*	30	100.0
*EAU risk category*	*Intermediate*	16	53.3
	*High*	14	46.6
	*Total*	30	100.0
**Pathology (post-operative) results**			
		*n*	%
*Grade group (ISUP)* ^**a**^	*1*	0	0.0
	*2*	12	40.0
	*3*	10	33.3
	*4*	2	6.7
	*5*	6	16.7
	Total	30	100.0
*Pathological tumour stage (pT)*	*pT2*	13	43.3
	*pT3a*	13	43.3
	*pT3b*	2	6.7
	*pT4a*	2	6.7
	Total	30	100.0

*IQR* interquartile range, *PSA* prostate-specific antigen, *MSKCC* Memorial Sloan Kettering Cancer Centre, *ISUP* International Society of Urological Pathology, *EAU* European Association of Urology

^a^ISUP definition: ISUP 1 = Gleason score 3 + 3 = 6, ISUP 2 = Gleason score 3 + 4 = 7a, ISUP 3 = Gleason score 4 + 3 = 7b, ISUP 4 = Gleason score 4 + 4 = 8/Gleason score 3 + 5 = 8 /Gleason score 5 + 3 = 8, ISUP 5 = Gleason score 4 + 5 = 9/Gleason score 5 + 4 = 9/Gleason score 5 + 5 = 10

### Accuracy of [^18^F]DCFPyL-PET/MRI and mpMRI to detect local clinically significant prostate cancer on a patient level

In the 30 included patients, 420 segments (i.e., 12 prostate segments + 2 seminal vesicle segments per patient) were evaluated in imaging and histopathological mapping studies. PCa was present in 143 of the 420 (33.8%) segments on histopathological examination, and csPCa was found in 142 of the 420 segments (33.5%) (median 5.5 segments per patient, IQR 4.0–6.8). A total of 29/30 (96.6%) patients showed increased PSMA-expression in the prostate on [^18^F]DCFPyL PET/MRI. The primary potential biopsy advice by the nuclear medicine physicians harboured csPCa in 27/30 (90.0%) patients and detected the index PCa lesion in 23/30 (76.6%) patients based on [^18^F]DCFPyL PET/MRI images. For mpMRI, 28/30 (93.3%) patients displayed a score of PI-RADS 3 or higher. The mpMRI-based primary potential biopsy advice by the radiologists harboured csPCa in 28/30 (93.3%) patients and detected the index PCa lesion in 23/30 (76.6%) patients. When both the primary and secondary advised segments would potentially be targeted for biopsy, both [^18^F]DCFPyL PET/MRI and mpMRI detected the index lesion in 27/30 (90.0%) patients. An example of potential [^18^F]DCFPyL PET/MRI**-**guided biopsy advice and concurrent histopathological examination of the RARP specimen is shown in Fig. 1, and an example of a discrepant imaging biopsy advice is shown in Supplement 1.

### Accuracy of [^18^F]DCFPyL PET/MRI and mpMRI to detect local clinically significant prostate cancer on a segmental level

The sensitivity, specificity, PPV, and NPV of [^18^F]DCFPyL PET/MRI to detect csPCa per segment with total agreement were 50.0% (95%CI 41.5–58.5), 89.9% (95%CI 85.7–91.6), 71.7% (95%CI 63.2–78.9), and 77.9% (95%CI 77.8–80.7), respectively (Table 2). For near-total agreement, the sensitivity, specificity, PPV, and NPV of [^18^F]DCFPyL PET/MRI to detect csPCa per segment were 80.3% (95%CI 72.8–86.5%), 95.3% (95%CI 92.1–97.5), 89.76% (95%CI 83.7–93.8), and 90.4% (95%CI 87.2–93.0), respectively.

**Table 2:** The diagnostic value of [^18^F]DCFPyL-PET/MRI for detecting PCa on a per-segment basis

Segment-based accuracy—Near-total agreement					
	PCa positive (histopathology)	PCa negative (histopathology)	Total	% (95% CI)	
PCa positive (PET/MRI)	114	13	127	89.8 (83.7 – 93.8)	PPV
PCa negative (PET/MRI)	28	265	293	90.4 (87.2 –93.0)	NPV
Total	142	278	420	33.8	Prevalence
% (95% CI)	80.3 (72.8 –86.5)	95.3 (92.1 –97.5)			
	Sensitivity	Specificity			
Segment-based accuracy—Total agreement					
	PCa positive (histopathology)	PCa negative (histopathology)	Total	% (95% CI)	
PCa positive (PET/MRI)	71	28	99	71.7 (63.2–78.9)	PPV
PCa negative (PET/MRI)	71	250	321	77.9 (74.8–80.7)	NPV
Total	142	278	420	33.81	Prevalence
% (95% CI)	50.0 (41.5–58.5)	89.9 (85.8–93.2)			
	Sensitivity	Specificity			

*PET* positron emission tomography, *MRI* magnetic resonance imaging, *PCa* prostate cancer, *CI* confidence interval, *PPV* positive predictive value, *NPV* negative predictive value

The sensitivity, specificity, PPV, and NPV of mpMRI alone to detect csPCa per segment with total agreement were 54.2% (95%CI 45.7–62.6), 94.2% (95%CI 90.8–96.7), 82.8% (95%CI 74.5–88.8), and 80.1% (95%CI 77.1–82.9), respectively (Table 3). For near-total agreement, the sensitivity, specificity, PPV, and NPV of mpMRI to detect csPCa per segment were 79.6% (95%CI 72.0–85.8), 98.6% (95%CI 96.3–99.61), 96.5% (95%CI 91.4–98.7), and 90.4% (95%CI 87.2–92.9), respectively.

**Table 3:** The diagnostic value of mpMRI for detecting PCa on a per-segment basis

Segment-based accuracy—Near total agreement					
	PCa positive (histopathology)	PCa negative (histopathology)	Total	% (95% CI)	
PCa positive (mpMRI)	113	4	117	96.6 91.4—98.7)	PPV
PCa negative (mpMRI)	29	274	303	90.4 (87.2–92.9)	NPV
Total	142	278	420	33.8	Prevalence
% (95% CI)	79.6 (72.0—85.9)	98.6 (96.4—99.6)			
	Sensitivity	Specificity			
Segment-based accuracy—Total agreement					
	PCa positive (histopathology)	PCa negative (histopathology)	Total	% (95% CI)	
PCa positive (mpMRI)	77	16	93	82.8 (74.5–88.8)	PPV
PCa negative (mpMRI)	65	262	327	80.12 (77.1–82.9)	NPV
Total	142	278	420	33.8	Prevalence
% (95% CI)	54.2 (45.7–62.6)	94.2 (90.8–96.7)			
	Sensitivity	Specificity			

*PET* positron emission tomography, *MRI* magnetic resonance imaging, *PCa* prostate cancer, *CI* confidence interval, *PPV* positive predictive value, *NPV* negative predictive value

**Fig. 2:**
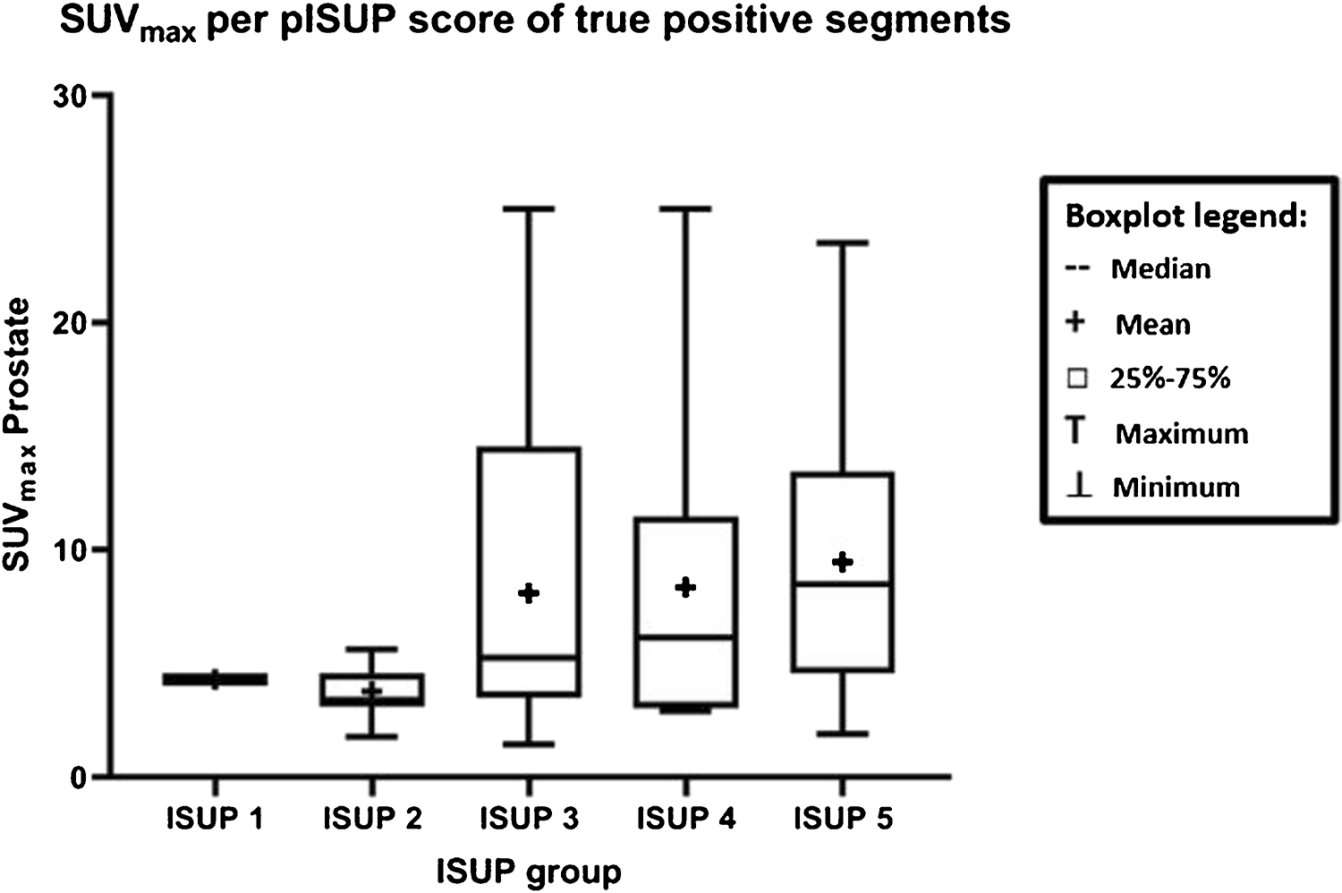
Distribution of SUV_max_ of [^18^F]DCFPyL-PET/MRI detected csPCa lesions per ISUP category. The SUV_max_ of detected lesions differed between ISUP grades (*p* = 0.02). ISUP Definition: ISUP 1 = Gleason score 3 + 3 = 6, ISUP 2 = Gleason score 3 + 4 = 7a, ISUP 3 = Gleason score 4 + 3 = 7b, ISUP 4 = Gleason score 4 + 4 = 8/Gleason score 3 + 5 = 8 /Gleason score 5 + 3 = 8, ISUP 5 = Gleason score 4 + 5 = 9/Gleason score 5 + 4 = 9/Gleason score 5 + 5 = 10

True positive segments measured on [^18^F]DCFPyL PET/MRI had a median SUV_max_ of 5.22 (IQR 3.38–8.5), which nearly significantly differs with the median SUV_max_ of false positive segments of 3.45 (IQR 2.76–7.17) (*p* = 0.058). The median SUV_max_ measured on [^18^F]DCFPyL PET/MRI of true positive segments was significantly different among ISUP grade groups (*p* = 0.02) (Fig. 2).

### Comparing the accuracy of [^18^F]DCFPyL PET/MRI vs. mpMRI to detect local prostate cancer on a segmental level

**Fig. 3:**
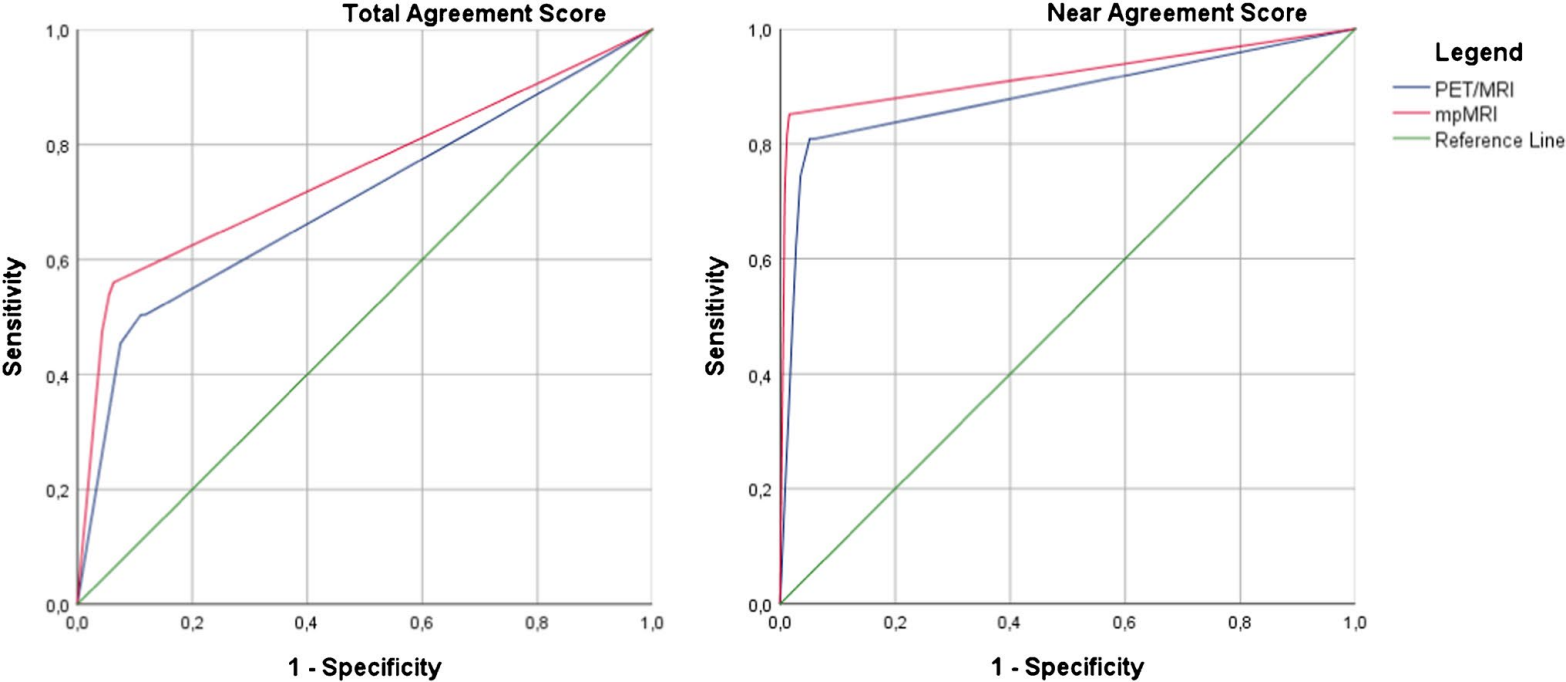
Receiver‐operating curves (ROC) for both [^18^F]DCFPyL-PET/MRI and mpMRI for the detection of clinically significant prostate cancer on a segment basis. Three categories were implemented: PSMA-RADS/PI-RADS 1–2 was labelled as negative, PSMA-RADS/PI-RADS 3 as equivocal, and PSMA-RADS/PI-RADS 4–5 as positive. The area under the ROC curve (AUC) for the total agreement scores was 0.70 (95%CI 0.64–0.76) for PET/MRI, and 0.75 (95%CI 0.70–0.81) for mpMRI (*p* = 0.15). The AUC for the near-total agreement scores was 0.87 (95%CI 0.83–0.91) for PET/MR, and 0.90 (95%CI 0.87–0.94) for mpMRI (*p* = 0.17)

The sensitivity of [^18^F]DCFPyL PET/MRI vs. mpMRI alone for detecting csPCa on a per-segment basis did not differ for the total agreement scores (Table 4). When analyzing the near-total agreement scores, in the ISUP ≥ 4 subgroup, the mpMRI performed better with a sensitivity of 95.7% vs. 76.6% of [^18^F]DCFPyL PET/MRI (*p* = 0.01). The sensitivities for the near-total agreement scores for the whole cohort and ISUP ≤ 3 subgroup did not differ statistically (*p* = 0.3 and *p* = 0.6, respectively). The specificity for the detection of csPCa was significantly higher for mpMRI vs. [^18^F]DCFPyL PET/MRI for both the total agreement (94.2% vs. 89.9%, respectively (*p* = 0.04)) and the near-total agreement scores (98.6% vs. 95.3%, respectively (*p* = 0.02)). The area under the ROC curve (AUC) of the detection of csPCa on a segment basis for the total agreement scores was 0.70 (95%CI 0.64–0.76) for [^18^F]DCFPyL PET/MRI, and 0.75 (95%CI 0.70–0.81) for mpMRI (*p* = 0.15). The AUC for the near-total agreement scores was 0.87 (95%CI 0.83–0.91) for [^18^F]DCFPyL PET/MRI, and 0.90 (95%CI 0.87–0.94) for mpMRI (*p* = 0.17) (Fig. 3).

**Table 4:** Comparing the sensitivity and specificity of [^18^F]DCFPyL-PET/MRI vs. mpMRI for the detection of PCa on a segment basis

Segment-based sensitivity and specificity of ^18^F-DCFPyL-PET/MRI vs. mpMRI			
	Sensitivity PET/MR % (95% CI)	Sensitivity mpMRI % (95% CI)	P
Detection of csPCA total agreement	50.0 (41.5 – 58.5)	54.2 (45.7 – 62.6)	0.3
Detection of csPCA near-total agreement	80.3 (72.8 – 86.5)	79.6 (72.0 – 85.9)	0.3
Detection of ISUP 2–3 total agreement	51.1 (40.5 – 61.5)	51.1 (40.5 – 61.5)	1.0
Detection of ISUP 2–3 near-total agreement	83.0 (73.8 – 90.0)	79.8 (70.3 – 87.4)	0.6
Detection of ISUP ≥ 4 total agreement	48.9 (34.1 – 63.9)	66.0 (50.7 – 79.1)	0.1
Detection of ISUP ≥ 4 near-total agreement	76.6 (62.0 – 87.7)	95.7 (85.5 – 99.5)	0.01
	Specificity PET/MR % (95% CI)	Specificity mpMRI % (95% CI)	*P*
Detection of csPCA total agreement	89.9(85.8 – 93.2)	94.2 (90.8 – 96.7)	0.04
Detection of csPCA near-total agreement	95.3 (92.1 –97.5)	98.6 (96.4 – 99.6)	0.02

*PET* positron emission tomography, *MRI* magnetic resonance imaging, *PCa* prostate cancer, *CI* confidence interval

^a^ ISUP definition: ISUP 1 = Gleason score 3 + 3 = 6, ISUP 2 = Gleason score 3 + 4 = 7a, ISUP 3 = Gleason score 4 + 3 = 7b, ISUP 4 = Gleason score 4 + 4 = 8/Gleason score 3 + 5 = 8 /Gleason score 5 + 3 = 8, ISUP 5 = Gleason score 4 + 5 = 9/Gleason score 5 + 4 = 9/Gleason score 5 + 5 = 10

### Accuracy of [^18^F]DCFPyL-PET/MRI and mpMRI to detect locally advanced prostate cancer

The final histopathological analysis revealed pT3a in 13/30 (43.3%) patients, pT3b in 2/30 (6.7%) patients, and pT4a in 2/30 (6.7%) patients. The sensitivity, specificity, PPV, and NPV of [^18^F]DCFPyL PET/MRI to detect locally advanced tumour growth (≥ pT3a) were 35.3% (95%CI 14.2–61.7), 84.6% (95%CI 54.6–98.1), 75.0% (95%CI 41.8–92.6), and 50.0% (95%CI 39.6–60.4), respectively (Table 5). For mpMRI, the sensitivity, specificity, PPV, and NPV to detect ≥ pT3a were 41.2% (95%CI 18.4–67.1), 100.0% (95%CI 75.3–100.0), 100.0% (95%CI n.a.), and 56.5% (95%CI 46.6–65.9), respectively (Table 6).

**Table 5:** The diagnostic value of [^18^F]DCFPyL-PET/MRI for the prediction of extraprostatic tumour growth (pT3a-b) in patients who underwent robot-assisted radical prostatectomy. Positive ^18^F-DCFPyL-PET/MRI findings for extracapsular extension (rT3a) and seminal vesicle invasion (rT3b) were compared to histopathological results (pT3a,b)

Locally advanced (pT3a-b vs. pT2)					
	pT3a-b	pT2	Total	% (95% CI)	
rT3a-b	6	2	8	75.0 (41.8–92.6)	PPV
rT2	11	11	22	50.0 (39.6– 60.4)	NPV
Total	17	13	30	56.7	Prevalence
% (95% CI)	35.3 (14.2–61.7)	84.6 (54.6– 98.1)			
	Sensitivity	Specificity			

*PET* positron emission tomography, *MRI* magnetic resonance imaging, *PCa* prostate cancer, *CI* confidence interval, *PPV* positive predictive value, *NPV* negative predictive value

**Table 6:** The diagnostic value of mpMRI for the prediction of extraprostatic growth (T3a-b) in patients who underwent robot-assisted radical prostatectomy. Positive mpMRI findings for extracapsular extension (rT3a) and seminal vesicle invasion (rT3b) were compared to histopathological results

Locally advanced (pT3a-b vs. pT2)					
	pT3a-b	pT2	Total	% (95% CI)	
rT3a-b	7	0	7	100.0	PPV
rT2	10	13	23	56.52 (46.6– 65.9)	NPV
Total	17	13	30	56.7	Prevalence
% (95% CI)	41.2 (18.4–67.1)	100.0 (75.3–100.0)			
	Sensitivity	Specificity			

*PET* positron emission tomography, *MRI* magnetic resonance imaging, *PCa* prostate cancer, *CI* confidence interval, *PPV* positive predictive value, *NPV* negative predictive value

### Intra-observer agreement

For all 420 segments, the intra-observer observed agreement (OA) for identifying csPCa on a per-segment basis using [^18^F]DCFPyL PET/MRI included 85% (95% CI 81.8–87.8), with a positive agreement (PA) of 67.7% (95% CI 58.7–75.2) and a negative agreement (NA) of 90.2% (95% CI 86.9–92.7). When using mpMRI, this OA involved 87.6% (95%CI 84.7–90.2), with a PA of 67.3% (95%CI 58.7–75.2) and a NA of 90.1% (95%CI 86.9–92.7). The OA for detecting locally advanced tumours using [^18^F]DCFPyL PET/MRI was 86.7% (95%CI 83.6–89.3), with a PA 66.7% (95%CI 57.3–75.2) and a NA of 91.7% (95%CI 88.8–94.0). The latter scores for mpMRI were 86.7% (95%CI 83.6–89.3), 75.0% (95%CI 67.4–81.6), and 91.7% (95%CI 88.8–94.0).

## Discussion

In this prospective study, the diagnostic accuracy of both [^18^F]DCFPyL PET/MRI and mpMRI imaging was compared for the ability to localize and stage csPCa within the prostate gland and to guide potential targeted prostate biopsies. A total of 30 patients with intermediate- to high-risk PCa were evaluated, all of whom received both imaging modalities before RARP. Imaging and histopathology findings on radical prostatectomy specimens were compared using a 14-segment prostate model. The targeted biopsy advice based on either [^18^F]DCFPyL PET/MRI or mpMRI detected the index csPCa lesion in 27/30 (90.0%) patients.

In contrast to the biopsy advice on a patient basis, both [^18^F]DCFPyL PET/MRI and mpMRI imaging demonstrated moderate sensitivity on a per segment basis for the detection of csPCa (50.0% and 54.2%, respectively (*p* = 0.32)), and improved with the near-total agreement score for both [^18^F]DCFPyL PET/MRI imaging and mpMRI (80.3% and 79.6%, respectively (*p* = 0.33)). The AUC of [^18^F]DCFPyL PET/MRI vs. mpMRI for detecting csPCa on segmental level did not differ for the total agreement scores (0.70 vs. 0.75, respectively (*p* = 0.15)) and near-total agreement scores (0.87 vs. 0.90, respectively (*p* = 0.17)). Moreover, the segment-based specificity for the detection of csPCa was significantly higher for mpMRI vs. [^18^F]DCFPyL PET/MRI for both the total agreement (94.2% vs. 89.9%, respectively (*p* = 0.04)) and the near-total agreement scores (98.6% vs. 95.3%, respectively (*p* = 0.02)). Based on these results, [^18^F]DCFPyL PET/MRI is comparable to mpMRI in the detection of csPCa on a segment level, and slightly underperforms for specificity.

Our study demonstrates that [^18^F]DCFPyL PET/MRI has the potential to target biopsies at a similar precision as mpMRI, and could possibly be used in a biopsy-naïve setting, since the per-patient detection of the index lesion and the AUC and sensitivity are comparable to the already established mpMRI. [^18^F]DCFPyL PET/MRI would have the advantage of total-body metastasis screening, which is not possible for mpMRI. One drawback could be the difference in specificity, which was significantly higher for mpMRI on a segment basis. But since [^18^F]DCFPyL PET/MRI still has an excellent specificity, we believe that it would still be sufficient for clinical practice.

Only one previous study has been published on diagnostic accuracy for detecting local PCa using [^18^F]DCFPyL PET/MRI. In this prospective case series by Bauman et al., all index PCa lesions of the 6 included patients were detected with both imaging modalities before RARP, which is in line with our results (90.0%) for both mpMRI and [^18^F]DCFPyL PET/MRI. However, all of these 6 patients had a GG of 2, which does not represent the full scope of high-risk PCa. Due to the study’s small sample size, no segment-based sensitivity and specificity analysis was performed. The complete series of 24 planned patients for this study will be published at a later stage, allowing for comparison with our findings.

Apart from the [^18^F]DCFPyL tracer employed in our study, similar studies using the more prevalent [^68^ Ga]Ga-PSMA-11 have been published [17]. In a retrospective series by Hicks et al., 32 patients were reviewed receiving a [^68^ Ga]Ga-PSMA-11 PET/MRI and a mpMRI prior to RARP [30]. Using a 30-segment mapping model and also implementing a similar total and a near-total agreement system, [^68^ Ga]Ga-PSMA-11 showed superior sensitivity vs. mpMRI for the detection of PCa (total agreement: 67% vs. 42%, respectively (*p* < 0.001), near-total agreement: 74% vs. 50%, respectively (*p* < 0.001)). Also the specificity for detecting PCa was higher when using mpMRI compared to [^68^ Ga]Ga-PSMA-11 PET/MRI (total agreement: 79% vs. 71% (*p* < 0.001), near-total agreement: 88% vs. 90% (*p* = 0.99)), which is in line with our results. However, due to the retrospective design and the lack of dual reader interpretation, the results of the latter study might be less comparable to our results. The discrepancy of results might also be explained by the fact that this study included GG 1 tumours in their analysis (35% of tumours), vs. our analysis which only encompassed csPCa (GG 2 or higher), due to an almost complete absence of GG 1 tumours (only 1 detected in our study). mpMRI is known to be underperforming at GG 1 grades significantly vs. GG 2 or higher [31], which could explain the significant underperformance of mpMRI in the study by Hicks et al. The very low incidence of GG 1 tumours in our study could be explained by the fact this study only selected intermediate- to high-risk patients for surgery.

In another similar series by Eiber et al., 55 patients were included who received a [^68^ Ga]Ga-PSMA-11 PET/MRI and a mpMRI prior to RARP [32]. Unlike our study, the mapping model of this study comprised a 6-segment model without using near-total agreement scores, and also included GG 1 tumours with a prevalence of 12.9%. The sensitivity and specificity of [^68^ Ga]Ga-PSMA-11 PET/MRI vs. mpMRI in the detection of PCa on a segment basis were 76% vs. 43% (*p* = 0.001) and 97% vs. 98% (*p* = not presented), respectively, clearly favouring the detection of PCa by [^68^ Ga]Ga-PSMA-11 PET/MRI. Overall, aforementioned [^68^ Ga]Ga-PSMA-11 studies seem to favour the detection of PCa using PET/MRI over mpMRI, which is not in line with our results. One explanation for the difference in results of these studies compared to the present one could be that our study used a PET/MRI scanner using sequential acquisition, vs. a simultaneous acquisition scanner used by Eiber et al. (Siemens Biograph mMR, Siemens Healthcare, Erlangen, Germany) and Hicks et al. (SIGNA PET/MR; GE Healthcare, Waukesha, USA) [30, 32]. Sequential acquisition has been described before as a method that can cause movement artefacts, and the misalignment of PET and MRI during co-registration, which might have influenced our PET/MRI anatomical correlation results [33, 34]. The sensitivities of mpMRI csPCa detection in our study and in the studies by Hicks et al. and Eiber et al. appear to be lower than the sensitivities presented in a large meta-analysis by De Rooij et al. (54%, 42%, 43%, respectively vs. a pooled sensitivity of 78%) [7]. This discrepancy can be attributed to the lesion-based used in the studies in the meta-analysis vs. the segment-based analysis used in the present study and the studies by Hicks et al. and Eiber et al. The studies in the meta-analysis also included prostate biopsy correlation instead of the RARP correlation used in the present study, which can be less accurate due to a sampling bias.

When assessing the local staging, the differentiation between localized (T2) and locally advanced disease (≥ T3) can be of clinical value for risk stratification and treatment planning (e.g., nerve-sparing for RARP). The ability of both [^18^F]DCFPyL PET/MRI and mpMRI to detect locally advanced disease was low with sensitivities of 35.29% and 41.18%, respectively. This under-staging for mpMRI and [^18^F]DCFPyL PET/MRI is in line with previous reports [17, 31]. One retrospective study by Muehlematter et al. on 40 consecutive patients receiving both [^68^ Ga]Ga-PSMA-11 PET/MRI and mpMRI prior to RARP reported on a patient-specific sensitivity of detecting locally advanced disease of 69% vs. 46% (*p* = 0.04) and a specificity of 67% and 75% (*p* = 0.19) [15]. Due to the low sensitivity for the detection of local advancement presented in literature and in our study, both mpMRI and PET/MRI are not reliable enough to exclude its presence. However, the PPV for detecting locally advanced disease was 100% for both imaging modalities in our study. Therefore, we encourage both nuclear medicine physicians and radiologists to report on extracapsular extension or seminal vesicle invasion specifically, if detected.

The present study has some limitations, despite its prospective nature. Firstly, since all patients were PCa-biopsy–confirmed patients, a selection bias might have occurred by the readers. A biopsy-naïve cohort might have shown different results due to false-positive screening of PCa. Moreover, the majority of patients were diagnosed using mpMRI targeted biopsy, which might favour results towards mpMRI. Secondly, because the RARP specimen is removed from the body, it changes in shape due to organ slicing and shrinking artefacts. Therefore, no exact anatomical correlation is possible, although a leniency was applied by calculating the near-total agreement score. In the end, the actual correlation between imaging and histopathology is probably equidistant between the near-total and total agreement scores.

## Conclusions

[^18^F]DCFPyL-PET/MRI is able to accurately localize csPCa on a per-patient basis using anatomical mapping studies based on histopathology in the RARP specimen. The vast majority of index csPCa lesions (90.0%) were detected by both [^18^F]DCFPyL-PET/MRI and mpMRI on a patient basis. Both [^18^F]DCFPyL-PET/MRI and mpMRI were partly able to detect csPCa on a per-segment basis, and to predict the final pathological tumour stage. [^18^F]DCFPyL-PET/MRI does not outperform regular mpMRI for local staging or local detection, or to guide targeted biopsies.

## Supplementary Information

Below is the link to the electronic supplementary material.

Supplementary file1 (JPG 88 KB)

## Data Availability

Data are available on request to the corresponding author.

## References

[1] Mottet N, van den Bergh RCN, Briers E, Van den Broeck T, Cumberbatch MG, de Santis M (2021). EAU-EANM-ESTRO-ESUR-SIOG Guidelines on Prostate Cancer-2020 Update. Part 1: screening, diagnosis, and local treatment with curative intent. Eur Urol..

[2] Mottet N, Bellmunt J, Briers E, Bolla M, Bourke L, Cornford P, et al. EAU – ESTRO – ESUR – SIOG guidelines on prostate cancer. Arnhem, The Netherlands.: EAU Guidelines Office.; 2020. p. Epub ahead of print.

[3] Bjurlin MA, Carter HB, Schellhammer P, Cookson MS, Gomella LG, Troyer D (2013). Optimization of initial prostate biopsy in clinical practice: sampling, labeling and specimen processing. J Urol.

[4] Lane BR, Zippe CD, Abouassaly R, Schoenfield L, Magi-Galluzzi C, Jones JS (2008). Saturation technique does not decrease cancer detection during followup after initial prostate biopsy. J Urol..

[5] Kasivisvanathan V, Rannikko AS, Borghi M, Panebianco V, Mynderse LA, Vaarala MH (2018). MRI-targeted or standard biopsy for prostate-cancer diagnosis. N Engl J Med.

[6] Cornford P, Bellmunt J, Bolla M, Briers E, De Santis M, Gross T, et al. EAU-ESTRO-SIOG guidelines on prostate cancer. Part II: Treatment of relapsing, metastatic, and castration-resistant prostate cancer. Eur Urol. 2016. 10.1016/j.eururo.2016.08.002. 10.1016/j.eururo.2016.08.002 27591931

[7] de Rooij M, Hamoen EH, Witjes JA, Barentsz JO, Rovers MM (2016). Accuracy of magnetic resonance imaging for local staging of prostate cancer: a diagnostic meta-analysis. Eur Urol.

[8] Fahmy O, Khairul-Asri MG, Hadi S, Gakis G, Stenzl A (2017). The role of radical prostatectomy and radiotherapy in treatment of locally advanced prostate cancer: a systematic review and meta-analysis. Urol Int.

[9] Sokoloff MH, Brendler CB (2001). Indications and contraindications for nerve-sparing radical prostatectomy. Urol Clin North Am.

[10] Bolla M, Van Tienhoven G, Warde P, Dubois JB, Mirimanoff RO, Storme G (2010). External irradiation with or without long-term androgen suppression for prostate cancer with high metastatic risk: 10-year results of an EORTC randomised study. Lancet Oncol.

[11] Perner S, Hofer MD, Kim R, Shah RB, Li H, Moller P (2007). Prostate-specific membrane antigen expression as a predictor of prostate cancer progression. Hum Pathol.

[12] Rowe SP, Gorin MA, Allaf ME, Pienta KJ, Tran PT, Pomper MG (2016). PET imaging of prostate-specific membrane antigen in prostate cancer: current state of the art and future challenges. Prostate Cancer Prostatic Dis.

[13] Afshar-Oromieh A, Zechmann CM, Malcher A, Eder M, Eisenhut M, Linhart HG (2014). Comparison of PET imaging with a (68)Ga-labelled PSMA ligand and (18)F-choline-based PET/CT for the diagnosis of recurrent prostate cancer. Eur J Nucl Med Mol Imaging.

[14] Arslan A, Karaarslan E, Güner AL, Sağlican Y, Tuna MB, Özişik O (2020). Comparison of MRI, PSMA PET/CT, and fusion PSMA PET/MRI for detection of clinically significant prostate cancer. J Comput Assist Tomogr.

[15] Muehlematter UJ, Burger IA, Becker AS, Schawkat K, Hötker AM, Reiner CS (2019). Diagnostic accuracy of multiparametric MRI versus (68)Ga-PSMA-11 PET/MRI for extracapsular extension and seminal vesicle invasion in patients with prostate cancer. Radiology.

[16] Bauman G, Martin P, Thiessen JD, Taylor R, Moussa M, Gaed M (2018). [(18)F]-DCFPyL positron emission tomography/magnetic resonance imaging for localization of dominant intraprostatic foci: first experience. Eur Urol Focus.

[17] Evangelista L, Zattoni F, Cassarino G, Artioli P, Cecchin D, Dal Moro F (2021). PET/MRI in prostate cancer: a systematic review and meta-analysis. Eur J Nucl Med Mol Imaging.

[18] D'Amico AV, Whittington R, Malkowicz SB, Schultz D, Blank K, Broderick GA (1998). Biochemical outcome after radical prostatectomy, external beam radiation therapy, or interstitial radiation therapy for clinically localized prostate cancer. JAMA.

[19] Rowe SP, Gage KL, Faraj SF, Macura KJ, Cornish TC, Gonzalez-Roibon N (2015). (1)(8)F-DCFBC PET/CT for PSMA-based detection and characterization of primary prostate cancer. J Nucl Med.

[20] Ravert HT, Holt DP, Chen Y, Mease RC, Fan H, Pomper MG (2016). An improved synthesis of the radiolabeled prostate-specific membrane antigen inhibitor, [(18) F]DCFPyL. J Labelled Comp Radiopharm.

[21] Weinreb JC, Barentsz JO, Choyke PL, Cornud F, Haider MA, Macura KJ (2016). PI-RADS Prostate Imaging - Reporting and Data System: 2015, Version 2. Eur Urol.

[22] Fanti S, Goffin K, Hadaschik BA, Herrmann K, Maurer T, MacLennan S (2021). Consensus statements on PSMA PET/CT response assessment criteria in prostate cancer. Eur J Nucl Med Mol Imaging.

[23] Ceci F, Oprea-Lager DE, Emmett L, Adam JA, Bomanji J, Czernin J (2021). E-PSMA: the EANM standardized reporting guidelines v10 for PSMA-PET. Eur J Nucl Med Mol Imaging..

[24] von Eyben FE, Kiljunen T, Kangasmaki A, Kairemo K, von Eyben R, Joensuu T (2016). Radiotherapy boost for the dominant intraprostatic cancer lesion-a systematic review and meta-analysis. Clin Genitourin Cancer.

[25] Bodar YJL, Koene BPF, Jansen BHE, Cysouw MCF, Meijer D, Hendrikse HNH (2021). Standardized uptake values are adequate measures of lesional (18)F-DCFPyL uptake in patients with low prostate cancer disease burden. J Nucl Med.

[26] Epstein JI, Egevad L, Amin MB, Delahunt B, Srigley JR, Humphrey PA (2016). The 2014 International Society of Urological Pathology (ISUP) Consensus Conference on Gleason Grading of Prostatic Carcinoma: definition of grading patterns and proposal for a new grading system. The American journal of surgical pathology.

[27] Kesch C, Vinsensia M, Radtke JP, Schlemmer HP, Heller M, Ellert E (2017). Intra-individual comparison of 18F-PSMA-1007-PET/CT, multi-parametric MRI and radical prostatectomy specimen in patients with primary prostate cancer - a retrospective, proof of concept study. J Nucl Med.

[28] Giesel FL, Sterzing F, Schlemmer HP, Holland-Letz T, Mier W, Rius M (2016). Intra-individual comparison of (68)Ga-PSMA-11-PET/CT and multi-parametric MR for imaging of primary prostate cancer. Eur J Nucl Med Mol Imaging.

[29] de Vet HC, Mokkink LB, Terwee CB, Hoekstra OS, Knol DL (2013). Clinicians are right not to like Cohen's kappa. BMJ.

[30] Hicks RM, Simko JP, Westphalen AC, Nguyen HG, Greene KL, Zhang L (2018). Diagnostic accuracy of (68)Ga-PSMA-11 PET/MRI compared with multiparametric MRI in the detection of prostate cancer. Radiology.

[31] Drost FH, Osses DF, Nieboer D, Steyerberg EW, Bangma CH, Roobol MJ (2019). Prostate MRI, with or without MRI-targeted biopsy, and systematic biopsy for detecting prostate cancer. The Cochrane database of systematic reviews..

[32] Eiber M, Weirich G, Holzapfel K, Souvatzoglou M, Haller B, Rauscher I (2016). Simultaneous (68)Ga-PSMA HBED-CC PET/MRI improves the localization of primary prostate cancer. Eur Urol.

[33] Herzog H, Lerche C (2016). Advances in clinical PET/MRI instrumentation. PET clinics.

[34] Oprea-Lager DE, Yaqub M, Pieters IC, Reinhard R, van Moorselaar RJ, van den Eertwegh AJ (2015). A clinical and experimental comparison of time of flight PET/MRI and PET/CT systems. Mol Imaging Biol.

