# Supplementary figures and images for: Prospective analysis of clinically significant prostate cancer detection with [18F]DCFPyL PET/MRI compared to multiparametric MRI: a comparison with the histopathology in the radical prostatectomy specimen, the ProStaPET study
Source: Eur J Nucl Med Mol Imaging. 2021 Nov 2;49(5):1731–42. doi: 10.1007/s00259-021-05604-9 (PMC8940822; doi:10.1007/s00259-021-05604-9)

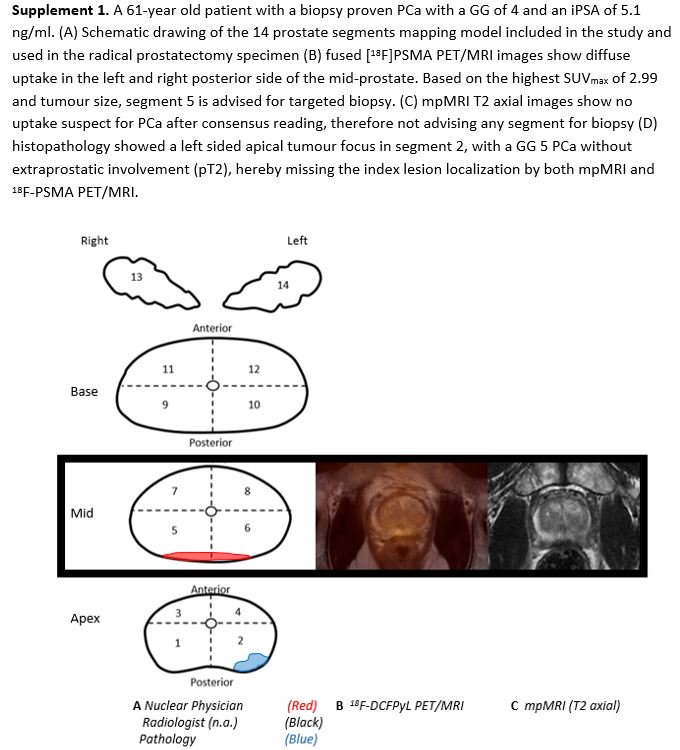

Supplement: Supplementary file 1 — Supplementary file1 (JPG 88 KB) [file 259_2021_5604_MOESM1_ESM.jpg]
